# Impact of civil war on emotion recognition: the denial of sadness in Sierra Leone

**DOI:** 10.3389/fpsyg.2013.00523

**Published:** 2013-09-03

**Authors:** Maria Allessandra Umiltà, Rachel Wood, Francesca Loffredo, Roberto Ravera, Vittorio Gallese

**Affiliations:** ^1^Section of Physiology, Department of Neuroscience, University of ParmaParma, Italy; ^2^Centre for Robotics and Neural Systems, School of Computing and Mathematics, University of PlymouthPlymouth, UK; ^3^Clinical Psychology Unit, Department of Psychology, ASL 1 (Azienda Sanitaria Locale) ImperieseImperia, Italy; ^4^S.C. Psicologia – ASL 1 (Azienda Sanitaria Locale) ImperieseImperia, Italy

**Keywords:** childhood, violence exposure, emotion recognition, young adults, war survivors, denial of sadness, Sierra Leone

## Abstract

Studies of children with atypical emotional experience demonstrate that childhood exposure to high levels of hostility and threat biases emotion perception. This study investigates emotion processing, in former child soldiers and non-combatant civilians. All participants have experienced prolonged violence exposure during childhood. The study, carried out in Sierra Leone, aimed to examine the effects of exposure to and forced participation in acts of extreme violence on the emotion processing of young adults war survivors. A total of 76 young, male adults (38 former child soldier survivors and 38 civilian survivors) were tested in order to assess participants' ability to identify four different facial emotion expressions from photographs and movies. Both groups were able to recognize facial expressions of emotion. However, despite their general ability to correctly identify facial emotions, participants showed a significant response bias in their recognition of sadness. Both former soldiers and civilians made more errors in identifying expressions of sadness than in the other three emotions and when mislabeling sadness participants most often described it as anger. Conversely, when making erroneous identifications of other emotions, participants were most likely to label the expressed emotion as sadness. In addition, while for three of the four emotions participants were better able to make a correct identification the greater the intensity of the expression, this pattern was not observed for sadness. During movies presentation the recognition of sadness was significantly worse for soldiers. While both former child soldiers and civilians were found to be able to identify facial emotions, a significant response bias in their attribution of negative emotions was observed. Such bias was particularly pronounced in former child soldiers. These findings point to a pervasive long-lasting effect of childhood exposure to violence on emotion processing in later life.

## Introduction

Emotion recognition requires the observer to relate the observed facial feature configurations to emotional states. Correct identification of an emotion involves the use of dynamic information about facial muscle movements to categorize emotional states and thence predict the behavior of other individuals (Ekman and Oster, 1979; Adelmann and Zajonc, 1989). Moreover, in ordinary social interactions the observer most likely does not wait until others' facial expression is at its peak to identify their emotional state. Rather, partial information available as an emotion expression is forming, provides cues to indicate the feelings of the observed person. Pollak and Sinha (2002) propose that rapid and accurate recognition of such early emotion signals facilitates social functioning and is guided by the observer's own experiences and emotion understanding.

As such, emotion recognition represents a complex developmental acquisition for the child. Studies of children with atypical emotional experience (e.g., those who have experienced maltreatment and abuse) demonstrate that early childhood exposure to high levels of hostility and threat biases emotion recognition such that children identify facial expressions of anger on the basis of less sensory input than controls (Pollak and Sinha, 2002; Pollak et al., 2009). Further evidence of an experiential bias in emotion recognition comes from data showing that abused children respond to faces expressing anger with relative increases in brain activity with respect to controls and that they demonstrate rapid orienting to and delayed disengagement from anger expressions regardless of modality of presentation (Shackman and Pollak, 2005; Pollak, 2008).

Masten et al. (2008) found that maltreated children, both with and without PTSD, displayed facilitated responses (faster reaction times compared to controls) when labeling emotion faces and that this effect was most pronounced with fear related stimuli. Interestingly, in this study there was no difference between maltreated groups (PTSD vs. non-PTSD) in emotion identification reaction times. Both displayed facilitated responses which were most pronounced with fearful faces.

Abuse and maltreatment of children most often occurs within the home or immediate family context (Tenney-Soeiro and Wilson, 2004). However, severe childhood trauma may also be associated with external events such as exposure to terrorism, societal breakdown and conflict. In a study of the effects of exposure to terrorism on children's emotion recognition Scrimin et al. (2009) found that compared to controls, exposed children were significantly more likely to label facial emotion morphs of sadness and anger expressions as anger. Exposed children were also found to display symptoms of depression (Moscardino et al., 2010). In this regard, studies of mental health and psychosocial functioning among refugees and other civilian survivors of war and mass violence indicate very high levels of traumatic experience and psychiatric sequelae (Roberts et al., 2008). In a study of adult refugees from the Sierra Leone war, Fox and Tang (2000) found extreme degrees of traumatic experience and significant mental health problems. In another study of the after effects of the Sierra Leone war de Jong et al. (2000) found that the conflict had affected almost all members of the population and that a very high proportion of those surveyed had scores indicating acute disturbance and severe post-traumatic stress disorder. Furthermore, a study conducted in Sierra Leone on children survivors showed that they experienced intrusive images, bad dreams, nightmares, and intense arousal symptoms (Gupta and Zimmer, 2008).

Research in countries affected by war and civil conflict indicates childhood exposure to mass violence to also be associated with high levels of PTSD and psychological distress, (Morgan and Behrendt, 2009). The question of how such extreme and traumatic experience might affect emotion processing in the general population and, more specifically, in children requires further investigation. In particular, there remains an open question about the effects on children of forced participation in mass violence. In a number of recent conflicts very large numbers of children have been abducted and compelled to take part in the commission of atrocities. “Evidence suggests that in most cases, children in the fighting forces were those who committed many atrocities. Some believed they were above the law and, above the ‘human-being world’ (as the children would say)” (Medeiros, 2007).

Although studies have documented a high prevalence of psychological trauma in former child soldiers (Klasen et al., 2010a,b), to date no research has been carried out specifically focusing on the emotional development, in adult life, of such children. In the present study we examined the effects of passive (civilians) and passive and active (former child soldiers) exposure, during childhood, to extreme trauma and mass violence on facial emotion recognition. A recent study was conducted in a group of “street-boys” in Sierra Leone with the aim to investigate if early aversive experiences could interfere with emotion recognition, facial mimicry, and autonomic regulation (Ardizzi et al., 2013). Results of this study suggested that early aversive experiences alter emotion recognition and facial mimicry of emotions.

The data of the present study were collected in Sierra Leone and to the best of our knowledge this is the first study specifically focused on emotion recognition in an African population following an extended period of civil war. The present study aims to investigate whether the exposure to mass violence during childhood affects the emotion recognition in early adult life.

### Historical background

The civil war in Sierra Leone began in 1991 and continued until 2002. Although the conflict primarily concerned Sierra Leone, neighboring regions were also involved. The war, instigated by the Revolutionary United Front (RUF) was funded through the trade in diamonds smuggled by the RUF to Liberia for sale on the international market. The conflict led to the widespread destruction of infrastructure (homes, schools, and hospitals) and the internal displacement of almost a quarter of the population (Richards, 1998; Keen, 2005). Hundreds of thousands of people became refugees. The fighting affected almost the entire population (Burman and McKay, 2007) and was characterized by extreme violations of human rights (including mutilation, murder, destruction of property and looting) on all sides (Burman and McKay, 2007). Child soldiers were widely used with estimates of the numbers involved in fighting ranging from 5–10,000 up to 48,000. The RUF were known to force children to commit atrocities against family members and neighbors as a means of indoctrination, thus destroying the child's social world. Child soldiers were repeatedly raped and forced to take drugs to reduce their inhibitions against committing violent acts (Burman and McKay, 2007). On returning to their home communities after the war former child soldiers were often excluded and remained socially isolated by the extreme nature of their experiences (Medeiros, 2007; Betancourt et al., 2010).

## Methods

### Participants

Participants were 76 young, male adults from Sierra Leone. Of these participants 38 were former child soldier survivors (soldier group) and 38 were civilian survivors of the civil war (civilian group). Participants with psychiatric or neurologic diseases, users of drugs and/or alcohol were excluded. The mean ages of the two groups were Soldiers: 22.36 SD ± 2.60; Civilians: 22.15 SD ± 2.62. Some former child soldiers were unable to give their exact date of birth. The study was conducted in partnership with The Family Homes Movement (FHM) a rehabilitation center for former child soldiers in Freetown, Sierra Leone, and the Sengbeh Pieh school in Lakka. The assessment battery and experimental conditions were administered in krio local language by two clinical psychologists (Roberto Ravera and Francesca Loffredo) aided by a local translator (Ernest Sesay).

Participants in the soldier group were contacted via the FHM and were tested at the St. Michael Community Center in Lakka. Participants in the civilian group were contacted through a school in their hometown of Lakka, ~15 km from Freetown. These latter participants were all tested at the school. Former child soldiers were found to be from villages all over Sierra Leone, some did not know their place of birth. All participants in the civilian group were enrolled in secondary level education, and had received primary education. The former child soldiers in the study had no further education beyond primary level.

Informed consent was obtained from all participants. The experimental protocol, and the modalities by which the informed consent was collected were approved by the Ethics Committee of ASL1 of Imperia, Italy and by the Ethics Committee of the Ministry of Health and Sanitation of the Republic of Sierra Leone.

### Experimental protocol

The protocol included two experiments. Experiment 1 (Static images) assessed participants' ability to identify facial emotion expressions from black and white photographs. Experiment 2 (Movies) assessed participants' ability to identify emotion expressions from black and white movies. Stimuli were constructed based on the Montreal Facial Displays of Emotion stimulus set (MSFDE), (Beaupré et al., 2000; Beaupre and Hess, 2005). All stimuli (images and videos) were presented in a pseudo-random order on a laptop computer using Matlab software (MathWorks Inc.) and the Psychophysics Toolbox extensions (Brainard, 1997; Pelli, 1997). Participants were seated at a desk on which the stimulus display laptop was placed at a distance of ~30 cms. Participants were asked to produce the vocal response. All experimental sessions were video recorded.

#### Experiment 1 (static images)

A set of 128 photographs of prototypical facial emotion expressions (32 happiness, 32 sadness, 32 anger, and 32 fear) was used. Emotion expressions were modeled by Asian, African, Hispanic, and Caucasian actors with an equal number of males and females.

**Figure 1:**
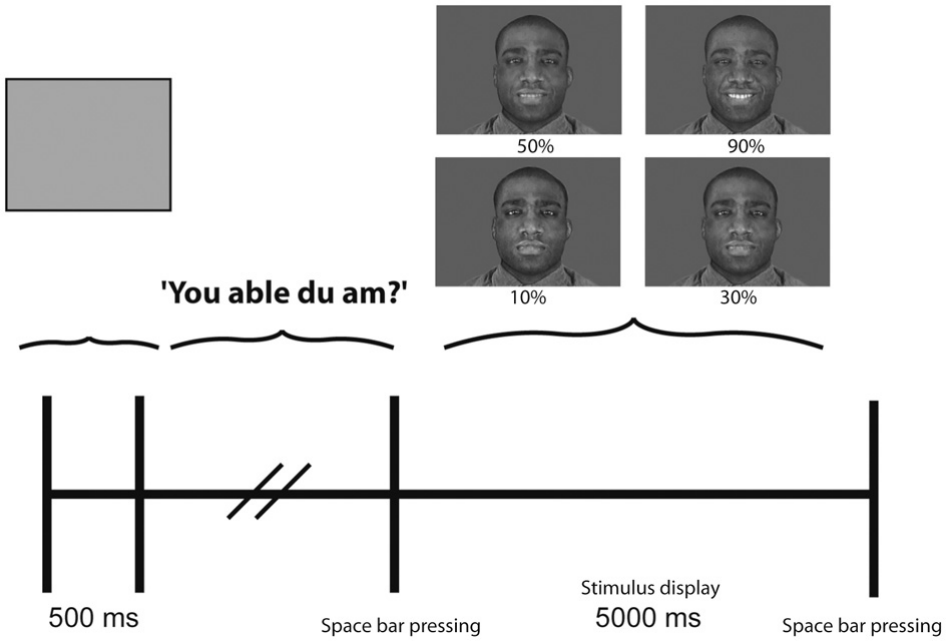
**Experimental paradigm.** The protocol for each trial was the following: (1) Blank screen 500 ms. (2) Appearance of the question “You able du am?” (Are you ready?) on screen until space bar pressed. (3) Stimulus presentation for 5000 ms (random presentation of single faces expressing different emotions at different intensities). (4) After space bar pressing, trial cycle repeats (i.e., 500 ms blank etc.). In the Movies experiment the experimental paradigm was exactly the same with the only difference that the stimulus presentation lasted 3000 ms.

For each emotion two exemplars from each ethnicity, one male and one female face, were presented. Emotion expressions ranged in intensity from near full expression to near neutral. The stimuli were obtained by morphing full expression images with the same face in a neutral posture to obtain a continuum of expression intensity. Images at 10, 30, 50, and 90% intensity were selected for presentation. Figure 1 shows the experimental paradigm together with an example of joy expressed at the 4 levels of intensity. The lower levels of expression were included to provide a number of ambiguous stimuli and to test participants' responses to less overt expressions. Fifty percent was selected in order to have a precise index of half of the expression intensity expressed in the stimuli. The particular increments used provide a gradient of expression intensity that robustly elicited a concomitant gradient in identification accuracy i.e., at 10% expression intensity participants performed around chance levels with accuracy increasing and peaking with 90% expression intensity. Thus, each emotion was expressed at four levels of intensity using male and female actors from the four ethnic groups described above. Stimuli were presented on screen for 5000 ms. The time interval was selected to ensure that participants' response accuracy was not affected by speed of presentation. Following presentation of each stimulus participants were required to verbally identify the emotion expressed in the image. Images were presented in a pseudo-random order as described above. The experimenter again recorded all participants' answers.

#### Experiment 2 (movies)

The stimulus set for this condition consisted of a series of 32 movies showing morphed transitions from neutral to full emotion expression (8 happiness, 8 sadness, 8 anger, and 8 fear). As before, emotions were modeled by actors from each of the four ethnic groups and numbers of male and female exemplars were balanced so that for each emotion an exemplar from each ethnic group and both gender was presented. Each movie lasted 3000 ms, this presentation length was determined by the length of the neutral to full expression morph and could not be altered without adversely affecting the realism of expression formation. Following each presentation a question mark appeared on the screen and participants were required to verbally identify the emotion displayed. As in the previous two experiments, movies were presented in a pseudo-random order and the experimenter recorded all participants' verbal answers. The stimuli and the experimental protocol were the same as those employed in the study of Ardizzi et al. (2013).

### Scoring

In all experiments each correct answer was given a score of 1 while incorrect answers and “don't know” responses were given a score of 0. In both experiments participants were given a forced choice of responses: happy, sad, angry, afraid in the Static Image and Movie experiments. All correct and incorrect answers were recorded for further statistical analyses.

### Data analysis

Statistical analyses were carried out on the data from the two Sierra Leone groups (Soldiers and Civilians). On the data collected from Experiment 1 (Static images) we performed a mixed designed ANOVA with Group, as between-factor, and Emotion (four levels) and Intensity (4 levels) as within factors.

In order to assess the percentage of correct responses we calculated the accuracy index of the two groups for each emotion (Wagner, 1993; Goeleven et al., 2008). A mixed-design ANOVA was conducted on the accuracy indexes with Group as between-factor and Emotion (Anger, Fear, Joy, Sadness) as within-factor.

Following the main analyses of performance by the two groups we conducted an additional analysis whereby we examined the errors made by participants (i.e., when making an incorrect emotion identification). We calculated the number of incorrect uses of each emotion label as a percentage of the total errors made by each subject. Thus, we calculated the number of times each emotion label occurred as an incorrect response and performed a mixed-design ANOVA with Group as between-factor and wrong Emotion label (Anger, Fear, Joy, Sadness) as within-factor.

In order to evaluate which wrong label was selected by participants, for each emotion, we calculated the number of incorrect use of each one of the other three emotion labels as a percentage of the total number of errors made by each subject for that emotion. Four separate mixed-design ANOVAs were performed on error rate with Group as between-factor and wrong Emotion label (3 levels) as within-factor.

Finally, in order to investigate the general response strategy used by the two groups of participants we calculated the number of times that both groups of participants labeled each emotion computing together correct and incorrect answers. A mixed-design ANOVA was conducted on total answers data with Group as between-factor and Emotion (Anger, Fear, Joy, Sadness) as within-factor.

On the data collected from Experiment 2 (Movies) we performed a mixed-design ANOVA with Group, as between-factor, and Emotion (Anger, Fear, Joy, Sadness) as within factors. An error analyses was also conducted also on data from the Movies experiment. In particular we examined the errors made by the two groups of participants when they made an incorrect identification of sadness. We performed this error analysis only with sadness presentation because with the other emotions the number of errors done by participant was not sufficient. We calculated the number of times each emotion label occurred as an incorrect response to the presentation of sadness as percentage of the total number of sadness presentations. A mixed-design ANOVA with Group as between-factor and Emotion (Anger, Fear, Joy) as within-factor was then carried out on this sadness error data.

Newman–Keuls *post-hoc* test was applied on all significant main factors and interactions.

Partial eta-squared was calculated for all significant main effects and interactions (Pierce et al., 2004).

## Results

### Experiment 1 (static images)

#### Correct answers analyses

Taking together all correct answers, mean scores for Experiment 1 were Soldiers: 59.55 ± SD 12.41, i.e., 46.52% correct identification; Civilians: 62.95 ± SD 16.52, i.e., 49.17% correct identification. In the first analysis of the emotion recognition data no group effect was found indicating that the two groups were not significantly different in their emotion recognition accuracy. Main effects of Emotion *F*_(3, 222)_ = 41.38 *p* < 0.001 (partial η^2^ = 0.35) and Intensity *F*_(3, 222)_ = 158.78 *p* < 0.001 (partial η^2^ = 0.68) plus an interaction between emotion and Intensity *F*_(9, 666)_ = 47.88 *p* < 0.001 (partial η^2^ = 0.39) were observed. This result indicates that participants' capacity to make accurate identification varied depending on the Emotion and the Intensity of the display. *Post-hoc* analysis carried out on the main effect of Emotion, using Newman–Keuls test showed that anger and fear had a similar recognition score (*p* > 0.05) and both were recognized significantly less than joy and sadness (*p*_*s*_ < 0.02). In addition, joy was the emotion with the significantly highest recognition score (*p*_*s*_ < 0.01) while recognition scores for joy and sadness were found to differ significantly between each other (*p*_*s*_ < 0.01).

**Figure 2:**
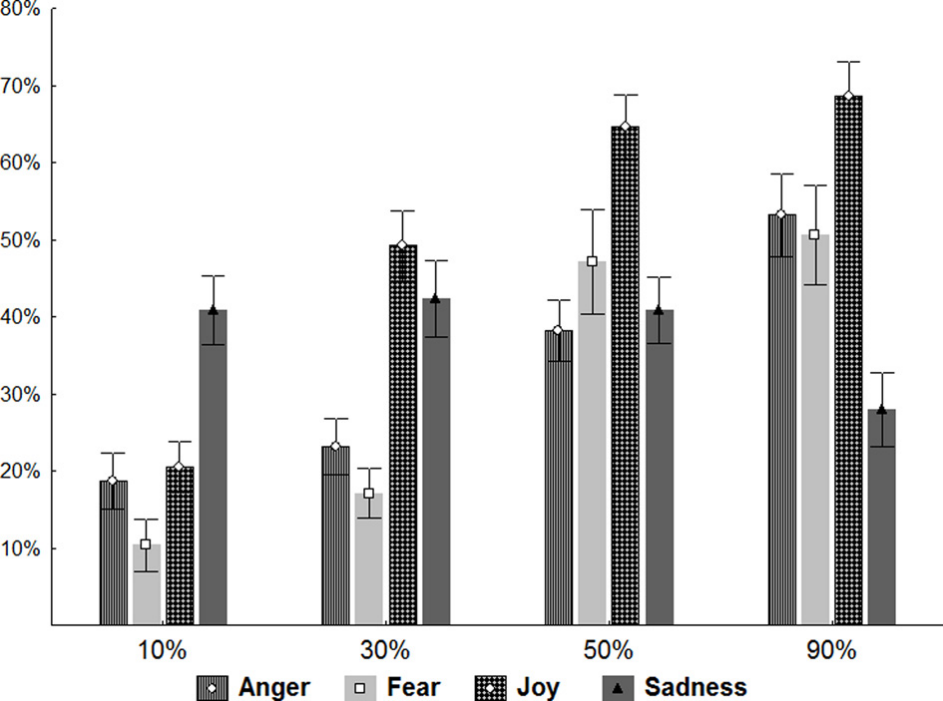
**Static Images.** Plots show the results of the mixed-design ANOVA performed on the correct answers given by the subjects of both groups of participants. The correct answers are expressed in percentage: 10, 30, 50, and 90% indicate the four levels of intensity of emotion expression.

*Post-hoc* analysis of the effect of emotion expression Intensity found significant differences between the 10, 30, and 50% levels of Intensity (*p* < 0.001), but not between 50 and 90%, indicating a plateau effect at 50%. *Post-hoc* analysis of the interaction between Emotion and Intensity (see Figure 2) showed that for all emotions except sadness, performance improved with increasing intensity of expression. For fear and joy, all levels of intensity significantly differed from each other (*p* < 0.001). With anger, performance did not differ significantly between 10 and 30% intensities, indicating a floor effect. However, between 30, 50, and 90%, the difference in performance was significant (*p* < 0.001). In the case of sadness a different pattern was observed. Here performance did not differ significantly between 10, 30, and 50% intensities. Most interestingly, performance on the 90% intensity stimuli was significantly worse than all the other levels. However, in the case of sadness performance was unaffected by intensity of expression up to 90% where it deteriorated significantly.

**Figure 3:**
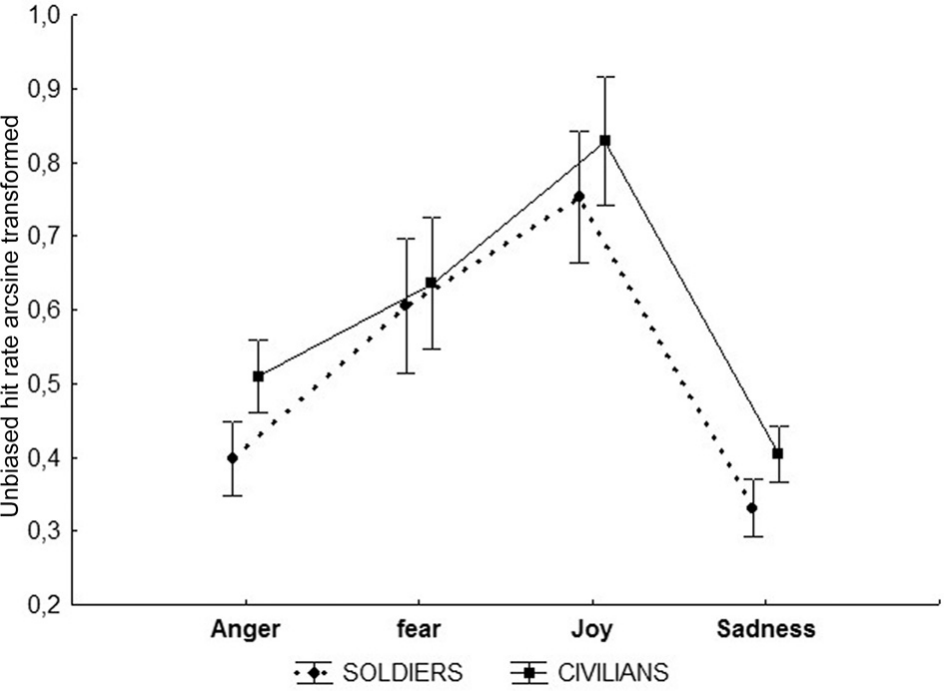
**Plots show the accuracy index of the two groups of participants for each emotion**.

The repeated-measures ANOVA conducted on the accuracy scores (Figure 3) showed a significant main effect of Group *F*_(1, 74)_ = 4.612 *p* < 0.04 (partial η^2^ = 0.05), and a significant main effect of Emotion *F*_(3, 222)_ = 75.813 *p* < 0.001 (partial η^2^ = 0.50), while the interaction between the two factors was not significant *F*_(3, 222)_ = 0.589 *p* > 0.05. Newman–Keuls *post-hoc* test applied on the main effect of Group showed that Civilians had a better accuracy performance than Soldiers (*p* < 0.04). *Post-hoc* comparisons applied to the main effect of Emotion indicated that the accuracy score was different for each emotion (all *p*_*s*_ < 0.005). The emotion labeled with less accuracy was sadness, followed by anger and fear. Finally, joy was the emotion recognized with the highest accuracy.

#### General analyses of errors

As an additional step we carried out an analysis of the errors made by participants; we calculated the number of incorrect uses of each emotion label as a percentage of the total errors made by each subject. We then performed a general analysis of the distribution of erroneous labels, i.e., what participants said when they made errors. Thus, we calculated the number of times each emotion label occurred as an incorrect response. We did not find a significant effect of Group; but we did find a main effect of Emotion *F*_(3, 222)_ = 100.82 *p* < 0.0001 (partial η^2^ = 0.57). We also found a significant interaction between Group and Emotion *F*_(3, 222)_ = 3.06 *p* < 0.02 (partial η^2^ = 0.04). *Post-hoc* analysis of the main effect of Emotion showed that when errors were made, fear and joy were attributed equally often (no significant difference) and less than sadness and anger. The distributions of incorrect sadness and anger labels were both different from all the other emotions, i.e., anger was attributed more often than fear and joy but less than sadness (*p*_*s*_ < 0.001) and sadness more frequently than all the other emotions (fear, joy, and anger *p*_*s*_ < 0.001).

**Figure 4:**
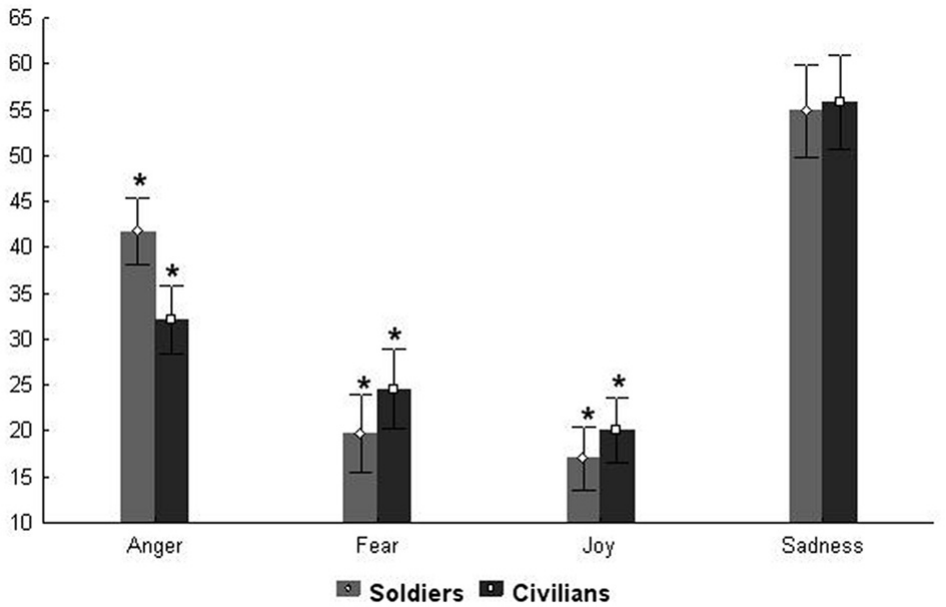
**Errors total Percentage.** Plots show the number of incorrect uses of each emotion label as a percentage (indicated in Y axis) of the total errors made by each subject of both groups of participants. ^*^Ps < 0.001.

Figure 4 shows the results of *post-hoc* analysis on the interaction Emotion × Group. This analysis revealed that soldiers and civilians differed in their erroneous use of anger, fear, and joy labels (*p* < 0.001). Both groups mistakenly applied sadness in the same way. This error data does not take into account the stimulus presented, only the label applied when a mistake was made. It is important to note that the first analysis (emotion recognition performance) reveals that the two groups did not significantly differ and that they both failed to recognize sadness, particularly at the highest levels of expression intensity (90%). While in the second analysis (errors) there was a significant difference between groups, except with respect to sadness, since both groups equally over attributed sadness when they made emotion identification errors.

#### Errors analyses emotion by emotion

**Figure 5:**
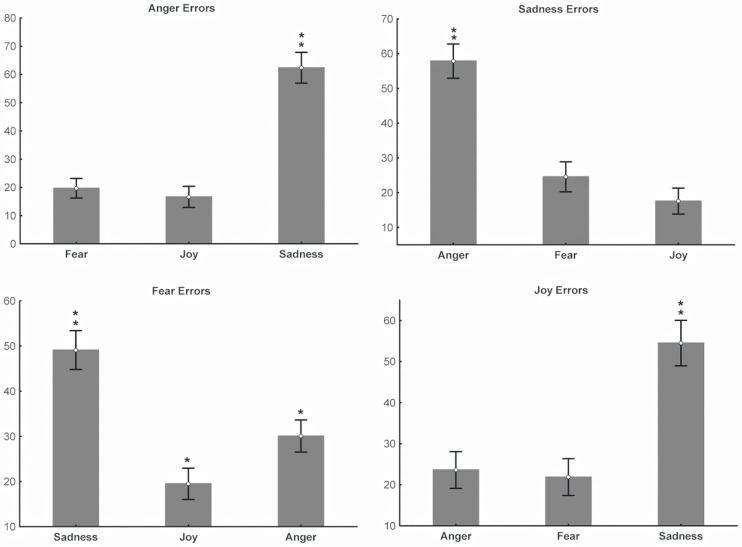
**Errors analyses for each emotion.** Plots show the results of the analyses of errors made by each subjects of both groups performed for each emotion with four separate ANOVAs. The number of incorrect uses of emotion labels is expressed as percentage (indicated in Y axis). **Anger**: ^**^ *ps* < 0.005; **Sadness**: ^**^ *ps* < 0.005; **Fear**: ^*^ *p* < 0.001, ^**^ *ps* < 0.005; **Joy**: ^**^ *ps* < 0.001.

We then carried out an analysis of errors emotion by emotion, in order to discover the relation between what participants saw and what they said when making mistakes. In general, when participants made incorrect emotion identification, they were most prone to say that the emotion they saw was sadness. We performed four separate mixed-design ANOVAs (one for each emotion) with Group as between-factor and wrong Emotion label (3 levels) as within-factor. These analyses revealed that when participants were presented with stimuli showing anger the two groups did not differ significantly in their use of erroneous labels but there was a main effect of Emotion *F*_(2, 148)_ = 97.27 *p* < 0.001 (partial η^2^ = 0.56). The *post-hoc* test on Emotion showed that both groups mislabeled anger as sadness. The mistaken use of the sadness label differed significantly from the use of joy and fear labels for these stimuli *p* < 0.001. The erroneous use of joy and fear when participants saw anger did not significantly differ (Figure 5 **Anger**).

When failing to recognize sadness the 2 groups did not differ but there was a main effect of Emotion *F*_(2, 148)_ = 64.48 *p* < 0.001 (partial η^2^ = 0.46). Both groups labeled sadness as anger significantly more often than fear or joy *p* < 0.001. Joy and fear did not differ (Figure 5 **Sadness**). In erroneous identifications of fear stimuli, again the groups did not differ. There was a main effect of Emotion *F*_(2, 148)_ = 43.56 *p* < 0.001 (partial η^2^ = 0.37) and sadness was the most commonly applied label. Here there is also a significant difference in the use of anger and joy as labels for fear stimuli; joy being used significantly less often than anger *p* < 0.001 (Figure 5 **Fear**). When participants made errors in recognizing joy [significant main effect of Emotion *F*_(2, 148)_ = 37.72 *p* < 0.001 with a partial η^2^ = 0.33] they labeled it as sadness significantly more than the other two emotions *p* < 0.001. Their use of anger and fear as labels for joy did not significantly differ (Figure 5 **Joy**).

#### Response strategy analysis

**Figure 6:**
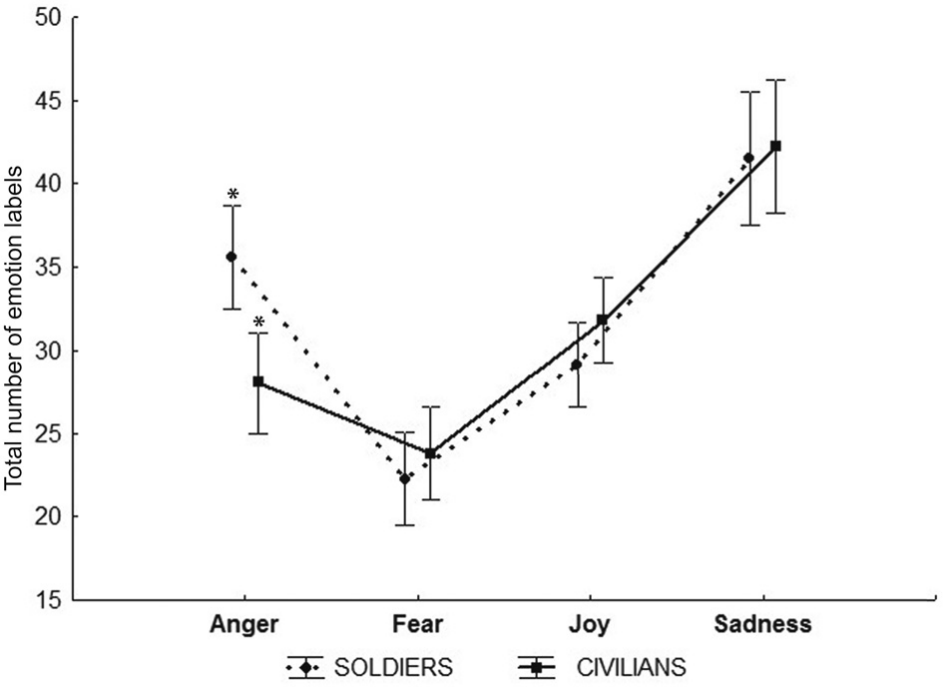
**Plots show the results of the Response strategy analysis that is the number of times that both groups of participants used a specific label to identify each emotion, summing together correct and incorrect answers.** ^*^p < 0.004.

In order to exclude the possibility that the mistaken use of the sadness label was due to a response strategy based on labeling each emotion a similar number of times, we calculated the number of times that both groups of participants used a specific label to identify each emotion, summing together correct and incorrect answers. Results of the mixed-design ANOVA (Figure 6) conducted on the total number of labels used for each emotion showed a significant main effect of Emotion *F*_(3, 222)_ = 38.742 *p* < 0.001 (partial η^2^ = 0.34) and a significant interaction Emotion × Group *F*_(3, 222)_ = 3.51 *p* < 0.02 (partial η^2^ = 0.04). The *post-hoc* test on Emotion showed that both groups did not use a general response strategy but, on the contrary, all emotions were differently labeled (*p* < 0.001) except for anger and joy (*p* > 0.04). Sadness label was used the highest number of times, while fear was the less used label. *Post-hoc* comparison applied to the significant Emotion × Group interaction showed that Soldiers group use significantly more frequently the label of anger with respect to Civilians (*p* < 0.004).

### Experiment 2 (movies)

#### Correct answer analyses

**Figure 7:**
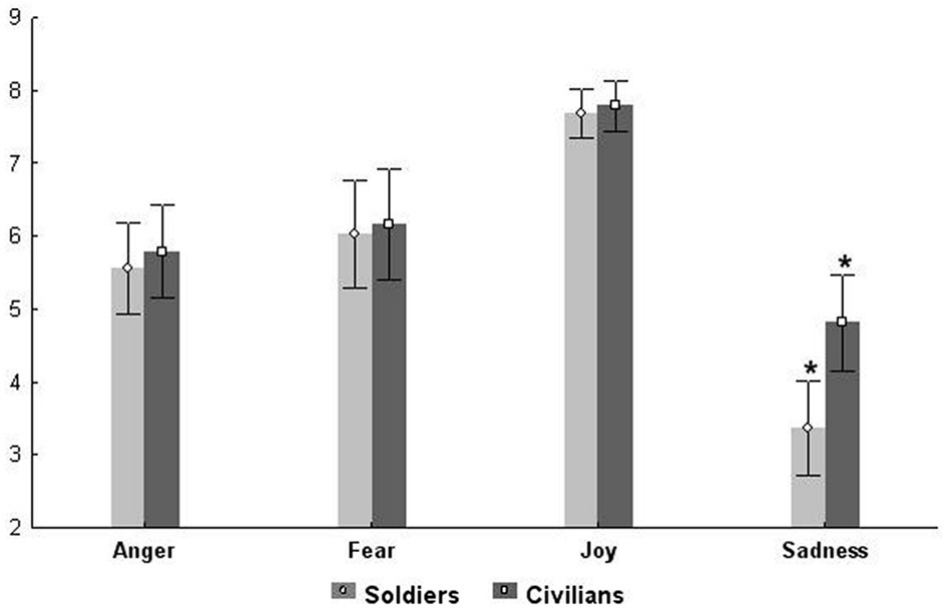
**Movies.** Plots show the results of the ANOVA performed on the correct answers given by both groups of participants. The correct answers are expressed in percentage (indicated in Y axis) and the values of 9 indicated the 90 percent of corrected responses. ^*^p < 0.01.

Mean scores for Experiment 2 (Movies) were Soldiers: 22.63 ± SD 4.37, i.e., 70.7% correct identification; Civilians: 23.89 ± SD 6.09, i.e., 74.6% correct identification. The main effect of Emotion was found to be significant *F*_(2, 219)_ = 55.44 *p* < 0.001 (partial η^2^ = 0.43) and the interaction of Group and Emotion was found to be marginally significant *F*_(2, 219)_ = 2.57 *p* = 0.054 (partial η^2^ = 0.03; Figure 7). *Post-hoc* analysis of factor Emotion revealed that joy differed significantly from the other emotions being the most accurately identified, while sadness differed from all the other emotions being the least accurately identified *p* < 0.001. Fear and anger did not differ. The *post-hoc* analysis of the Emotion × Group interaction showed that the two groups differed only on their performance in sadness recognition *p* < 0.01, significantly worse for soldiers.

#### Analyses of errors

**Figure 8:**
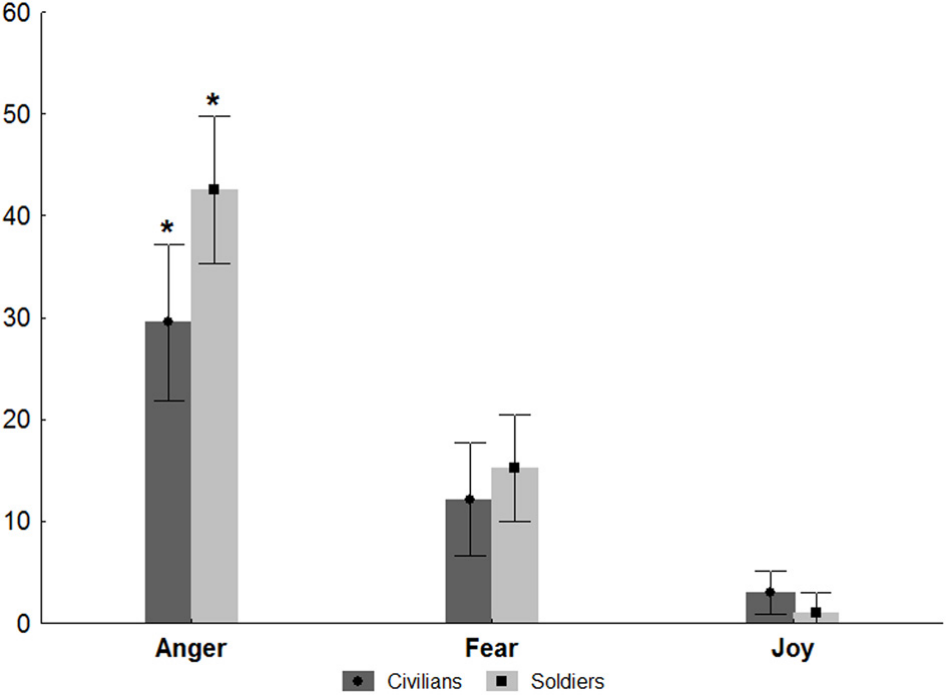
**Error analysis of sadness (movies).** Plots show the results of the ANOVA performed on the number of times each emotion label occurred as an incorrect response to the presentation of sadness. The incorrect responses are expressed as percentage (indicated in Y axis). ^*^p < 0.005.

The performance of both soldiers and civilians for recognition of dynamic stimuli (movies) reached an average of greater than 70% correct answers and thus the number of errors was not sufficient to perform error analyses similar to those applied on static images. However, because sadness was the emotion least accurately identified and soldiers differed from civilians only on their performance in sadness recognition, we then carried out an analysis of errors in order to discover what participants said when making mistakes in response to movies showing sadness. We calculated the number of times each emotion label occurred as an incorrect response to the presentation of sadness as percentage of the total number of sadness presentations. (see Figure 8).

The results of the ANOVA showed significant main effects of Group [*F*_(1, 68)_ = 6.28 *p* < 0.02] (partial η^2^ = 0.08) and Emotion [*F*_(2, 136)_ = 70.24 *p* < 0.001 (partial η^2^ = 0.50)]. In addition, the interaction of Group and Emotion was found to be significant *F*_(2, 136)_ = 3.43 *p* < 0.04 (partial η^2^ = 0.04). *Post-hoc* analysis of factor Group revealed that Soldiers made significantly more errors than Civilians (*p* < 0.02). The *post-hoc* test on Emotion showed that mistaken use of the anger label was significantly more common than erroneous use of fear and joy (*p*_*s*_ < 0.001) and that joy was the erroneous emotion label applied least often (*p*_*s*_ < 0.001).

Finally, results of Newman–Keuls test applied on the Emotion × Group interaction showed that anger was the most applied emotion by both groups of participants (*p*_*s*_ < 0.005). More interestingly, Soldiers and Civilians differed in their erroneous use of anger with soldiers using anger when erroneously labeling other emotions significantly more often than civilians (*p* < 0.005).

## Discussion

The aim of this study was to assess whether previous childhood exposure to extreme violence, either as a child soldier or a non-combatant civilian, has an effect on the ability to recognize facial emotion expressions in young adulthood. The civilian and former child-soldier participants in this study are now young adults but they were all children during the civil war in Sierra Leone (1991–2002). Child soldiers were exposed to extreme levels of violence and personal danger. They often were forced to participate in acts of violent aggression toward others, including members of their own family. Non-combatant children from Sierra Leone were also exposed to extreme violence and peril. Many saw members of their own family killed during the fightings. Both groups can reasonably be described as having experienced extreme and prolonged childhood trauma, the main difference between them being the degree to which they were forced to take an active role in the violence.

We found that both former child soldiers and civilians were able to recognize facial emotion expressions and that the performance of the two groups was statistically similar. Participants were able to match facial emotion expressions to the correct label more accurately with movies than with static images. This finding is in accord with previous facial emotion recognition data (Trautmann et al., 2009). However, despite participants demonstrating robust emotion recognition, both with static images and movies, they showed a strong bias with respect to sadness, which they failed to recognize. With movies, former child-soldiers were significantly worse than civilian participants in sadness recognition. In addition, despite there being a positive correlation between the accuracy of emotion identification and the intensity of expression for joy, fear, and anger, this was not the case for expressions of sadness. Both former soldiers and civilians made more errors in identifying facial expressions of sadness than in the other three emotions tested, and their performance deteriorated when sadness was shown at its maximal level of expression. When incorrectly labeling expressions of sadness both groups were most likely to label them as anger. Conversely, when mislabeling anger, both Sierra Leone groups most often identified it as sadness. This pattern was also found for mistaken identifications of joy and fear expressions. Thus, when seeing expressions of sadness former child soldiers and civilians were most likely to mislabel them as anger, whereas when mistaking other emotions, these participants were most likely to label them as sadness.

When making mistakes, the two Sierra Leone groups did differ in that former child soldiers were more likely to use anger when incorrectly labeling stimuli, while civilians were most likely to use the fear label. Both groups didn't show a global response strategy inducing the use of the four emotion labels a similar number of times but, on the contrary, the number of times each emotion label was used was specific for each emotion. Our data on former child soldiers show a response bias for the attribution of anger that has also been observed in children exposed to terrorism (Scrimin et al., 2009) and children subjected to physical abuse and maltreatment (Pollak and Sinha, 2002; Pollak and Tolley-Schell, 2003). However, in our data the bias for anger attribution was evident in erroneous applications of the label, whereas in the previous studies a greater degree of anger recognition accuracy was observed.

Furthermore, unlike children exposed to terrorism, who proved to be as proficient in identifying sadness as other basic emotions (Pollak and Sinha, 2002), the Sierra Leone participants in our study produced fewer than expected correct answers in response to stimuli expressing sadness. To our knowledge this result has not previously been demonstrated in survivors of childhood trauma war. This indicates that while war trauma may have particular characteristics, which determine the specific profile of its effects on the emotion processing of survivors still, there are commonalities with other forms of childhood maltreatment. Probably war trauma is not the same as any other kind of child abuse. It is certainly clear that emotion processing is one cognitive function which is deeply affected by such experiences in early life and that this justifies further research and should also guide therapeutic approaches aimed at ameliorating the effects of childhood maltreatment.

Our findings do not indicate a significant difference between the two Sierra Leone groups for emotion recognition with static images. However, a significant difference was observed with dynamic stimuli (i.e., movies). Soldiers and Civilians differed in their erroneous use of anger with soldiers using anger when erroneously labeling other emotions significantly more often than civilians. The same erroneous use of anger was recently demonstrated with a facial emotion recognition task conducted in Sierra Leone in a group of “street-boys” and in an age-matched control group. This study employed the same movies used in our experiment and their results showed that street boys group erroneously used the anger label significantly more often than other labels and than the control group. Participants of this study were younger (mean age 15 years) than our participants and this could be a possible explanation for the lack of bias in sadness recognition that was not found among street boys and that indeed characterized our study. Another possible reason could be the intensity of the traumatic events suffered during the civil war by both groups of our participants, the impact of this trauma could me much more intense than that experienced by street boys.

The lack of a strong difference in performance with static images is understandable when we take into account the extreme trauma likely to have been suffered by both groups. War (especially civil war) can lead to break down in civil order affecting the entire population. Individuals experience feelings of threat, witness extreme violence and may suffer separation from family and community (Dahl et al., 1998). Child soldiers in Sierra Leone were subjected to all of these experiences, however, it is highly likely that members of the civilian population also endured similar trauma. Ramsay et al. (1993) state that the purpose of torture is to produce psychological change through coercion, repression, punishment, or humiliation. All participants in this study can be said to have suffered experiences associated with torture and/or to have witnessed such treatment of others (most often members of their own family or close community) during early to middle childhood. It is interesting that a significant performance difference between the two groups was found with dynamic emotion stimuli. These findings suggest that further investigation of why dynamic stimuli are more “potent” in eliciting a differential response would be useful. A parsimonious but plausible explanation for the effectiveness of dynamic stimuli we found we propose could be that they better reflect a biological situation because in the real world humans interact with people in motion. In addition, the two groups had a reduced experience in exposition to digitized images presented on a computer screen and this could be the reason why the static images we presented were less able to elicit differences between the two groups.

To our knowledge no other study of emotion processing has ever been attempted in this population. There are very significant practical difficulties associated with studying former child soldiers and their non-combatant cohorts. A recent study compared the mental health status of former child soldiers with that of children who have never been conscripts of armed groups. The results showed that all participants experienced at least one type of trauma, but the majority of them experienced more than one traumatic event (Okello et al., 2007; Kohrt et al., 2008). The same studies also show that the prolonged exposure to traumas caused complex psychological alterations to all children.

Another study, conducted with a population of adolescent war survivors in Uganda aimed to correlate cognitive emotion regulation strategies, post-traumatic stress (PTS), and Internalizing vs. Externalizing symptoms (Amone-P'Olak et al., 2007). This study showed a significant positive relationship between denial of emotion and outcome measures such that the greater the degree of denial the higher participants scored on measures of PTS. Interestingly, the authors claimed that denial is a common cognitive strategy in trauma survivors.

The age at which all of our participants from Sierra Leone were exposed to extreme trauma and violence, ranged on average between 3 and 14 years. The results show that a systematic exposure to trauma for a substantial part of childhood produces long-lasting effects in adult life on how emotions are perceived and recognized. Furthermore, the results show that there is a relation between the intensity of exposure to trauma and the severity of emotional impairment, as shown by the emotion recognition performance and error analysis for the experiment with videos.

Terr (1991) proposed a framework for childhood trauma that distinguishes between single-incident trauma (Type 1) and repeated or prolonged trauma (Type II). In our view, all participants in this study could be considered as children that have been exposed to prolonged and repeated traumatic events. What is interesting is the fact that the same author identified unremitting sadness and denial as phenomena particularly evident in children exposed to prolonged trauma. Terr (1991) considered denial of their traumatic experiences to be a defensive mechanism used by children repeatedly exposed to extreme aversive events. At the same time, in her view, such Type II traumas leave the child in a state of “unremitting” sadness. The results of our study show a mechanism of denial at work in both populations of traumatized young adults war survivors. With movies such problem was significantly worse in former child-soldiers than in civilians. However, here we show denial applied not to the traumatic events themselves, but rather to an emotional state associated with and caused by those events. Ten years of exposure to civil war, more than half of our participants' lives, most likely make it almost impossible to deny the traumas endured. As a consequence, the only available resilience mechanism consists in denying sadness, the emotional consequence of those traumatic events. Hence, the same emotion must be denied when witnessed in others. In fact, both groups displayed greater denial the more sadness they saw. Interestingly, they were able to label the facial expression of an emotion as sadness when sadness was not expressed, as when mislabeling other emotions. May be a displacement, as a defensive mechanism, could be speculated, aiming to allow these young adults to admit also sadness in their experience in a more tolerable condition.

In the present study, the recognition deficit observed with both traumatized groups was mainly confined to facial expressions of sadness. Further research is required to establish the functional basis for participants' failure to identify sadness and for their tendency to mislabel this particular emotion. Previous studies have shown that childhood exposure to terrorism and collective violence is a potent risk factor for the development of psychological disorders including cognitive impairments and deficits in emotional and social functioning (Okello et al., 2007; Kohrt et al., 2008; Klasen et al., 2010a,b). All young adult survivors of the civil war In Sierra Leone, both ex-child soldiers and their non-combatant cohort, are thus at risk. These individuals are already caregivers to the next generation of young children growing up there. Since these individuals have clear difficulties in correctly assessing the facial expression of emotions in others, the results of our study suggest that the generations of the future are thus also at risk.

### Conflict of interest statement

The authors declare that the research was conducted in the absence of any commercial or financial relationships that could be construed as a potential conflict of interest.
